# Nutrient availability drives local seasonal movements of an endangered marine megafauna species

**DOI:** 10.1038/s41598-026-38138-x

**Published:** 2026-02-05

**Authors:** Daire Carroll, Irthisham Hassan Zareer, Clara Cánovas Pérez, Jessica Harvey-Carroll

**Affiliations:** 1 Department of Biology and Environmental Sciences, University of Gothenburg, Gothenburg, Sweden; 2 Gothenburg Global Biodiversity Centre, Gothenburg, Sweden; 3 Maldives Whale Shark Research (MWSR), H. Kaneerumage, Malé, Maldives

**Keywords:** Seasonal rhythms, Remote sensing, Marine megafauna, Animal behaviour, Conservation, Marine protected areas, Ecology, Ecology, Ocean sciences

## Abstract

Understanding drivers of animal movement is key to predicting species distributions and guiding conservation. Whale sharks (*Rhincodon typus*) are an endangered species known for broad seasonal migrations across oceans influenced by factors like temperature and prey availability. However, finer-scale local movements within aggregations are less studied. We analysed four years (2016–2019) of sightings data from a year-round aggregation in South Ari Marine Protected Area (SAMPA), the Maldives. Using MODIS-Aqua remote sensing data, we examined seasonal patterns in chlorophyll-a (Chl-a) and sea surface temperature (SST). Generalised additive mixed models (GAMMs) revealed significant seasonal rhythms in SST across SAMPA, and significant seasonal Chl-a variation in the south but not the east of the MPA. In a separate GAMM, we found that seasonal rhythms in shark sightings were significant throughout the MPA but more pronounced in the south than in the east. Chl-a was significantly associated with sightings, with both peaking in the south during the Northeast Monsoon (January–March). SST was not significantly associated with sightings. As Chl-a is tightly linked to the abundance of zooplankton, these findings suggest that whale shark movement within the year-round aggregation is driven by prey availability. The results could be used to inform dynamic management or predict aggregations elsewhere and responses to environmental change.

**Supplementary Information:**

The online version contains supplementary material available at 10.1038/s41598-026-38138-x.

## Introduction

Understanding both regional (e.g., at the ocean level) and local (e.g., within tens of kilometres) drivers of wildlife movement is essential for accurately predicting patterns of distribution and potential threats to species^1–3^. Many marine species move over vast distances in order to exploit seasonally abundant food sources or optimal climatic conditions^4–6^. Examples include the migrations of humpback whales (*Megaptera novaeangliae*), which travel thousands of kilometres between high-latitude feeding grounds and tropical breeding areas^7^, and extensive foraging trips made by the wandering albatross (*Diomedea exulans*) around the Southern Ocean^8^. Often, such movements follow predictable seasonal rhythms^4–6^ and overlap with human activities, putting them at risk of disruption^9,10^. On a finer-scale, animals move within their local environment for reasons such as foraging, cleaning, thermoregulation, predator avoidance, or in response to competition. Such local movement also often follows predictable daily or seasonal rhythms^1,11,12^. Integrating insights across spatial scales can provide a holistic understanding of animal behaviour, improving the effectiveness of conservation strategies and supporting the development of adaptive management approaches^3,13,14^.

The whale shark (*Rhincodon typus*) is the largest extant fish species. The species feeds primarily on plankton, small fish, and microscopic organisms^15–17^. Whale sharks are broadly distributed across tropical and warm-temperate oceans. They are highly migratory, with individuals undertaking extensive movements across ocean basins^4,5^. Many aspects of whale shark life history, including breeding locations and population connectivity, remain poorly understood^5,18^. Whale sharks are classified as Endangered by the IUCN Red List primarily due to a number of anthropogenic threats including overharvesting, bycatch, vessel strike, oil spills, plastic pollution, and stress from tourism^18–22^.

Whale sharks aggregate in a number of locations around the world. The majority of these aggregations are seasonal, with a small number being year-round^23,24^. Within aggregations, a high level of philopatric behaviour is observed^18,25,26^. The movement and aggregation patterns of whale sharks are driven by a combination of biological, environmental, and anthropogenic factors^4,23^. Aggregations often occur seasonally in areas with predictable prey availability, typically linked to zooplankton blooms, spawning events, or upwelling systems^27–32^. Temperature and bathymetry have also been identified as important environmental drivers of aggregations^11,33–35^. Migrations may be influenced by reproductive behaviour, although this is poorly understood in whale sharks and specific cues triggering long-distance movements are not known^23,36^. Human activities such as vessel traffic, harvesting, and bycatch likely impact aggregation sites^9,21,22,35^. Given the clear link between sea surface temperature (SST), prey abundance, and whale shark presence, it is likely that climate change will lead to shifts in the timing and locations of aggregations in the future^33,34^.

Less is known about local movement patterns of whale sharks within aggregations. The location of whale sharks within seasonal aggregations in the Azores and in the Caribbean Sea has been found to reflect prey abundance^37–39^. Whale shark presence in a year-round aggregation in Saleh Bay, Indonesia, was found to be positively associated with SST and chlorophyll-a concentration (Chl-a) during a single season (February to March)^40^.

In the Maldives, whale sharks are considered nationally important marine fauna^41–43^. Robust population estimates are limited and a high level of connectivity with the wider Indian Ocean is suspected^4^. Whale sharks are an important component the country’s shark tourism industry, valued at US$ 14.4 million^42,43^. While whale sharks are sighted in the waters around several atolls, tourism is focused on the southern tip of the Ari Atoll, where a globally rare year-round aggregation is found^18,41,42^. The aggregation consists predominantly of juvenile males, and may function as a secondary nursery^44–46^. Due to frequent whale shark sightings, the area was designated the South Ari Marine Protected Area (SAMPA) in 2009 but only received its first gazetted management plan in February 2025^47^. Until then, enforcement of regulations to protect whale sharks, such as limits to the number of vessels present during encounters, was limited. High predictability of sightings and easy access, combined with weak enforcement of regulations, have driven growing tourism pressure. As a result, over 60% of whale sharks in SAMPA have sustained major injuries^18^. Consequently, research has largely focused on managing human impacts rather than the biological drivers of whale shark presence and abundance.

The Maldives experience two distinct seasons: the Northeast Monsoon from January to March, characterised by dry weather and calm seas, and the Southwest Monsoon from mid-May to November, characterised by strong winds, increased rainfall, and rougher seas. April, early May, and December are considered transition periods^41,48^. Seasonal movements within SAMPA are thought to be linked to prey abundance, though this has not previously been quantitatively examined^11,41^.

Environmental drivers of habitat selection and aggregation of whale sharks on an ocean- or global-scale have been the subject of extensive research^4,34^. Local drivers within aggregations have been far less explored^11,41^. Afonso et al. (2014) suggested that, while abiotic factors such as temperature likely drive broad-scale trends, the distributions of sharks within aggregations are more likely to be driven by prey abundance^38^. To test this hypothesis, we compiled SST and Chl-a values as well as whale shark sightings from boat-based transect surveys for the year-round aggregation in SAMPA between the years 2016 and 2019. We tested for both seasonal variation and association between the number of sightings per survey and environmental variables. The results are highly relevant for the design of dynamic management strategies and marine protected areas for this and other endangered species.

## Methods

### Study site

**Fig. 1:**
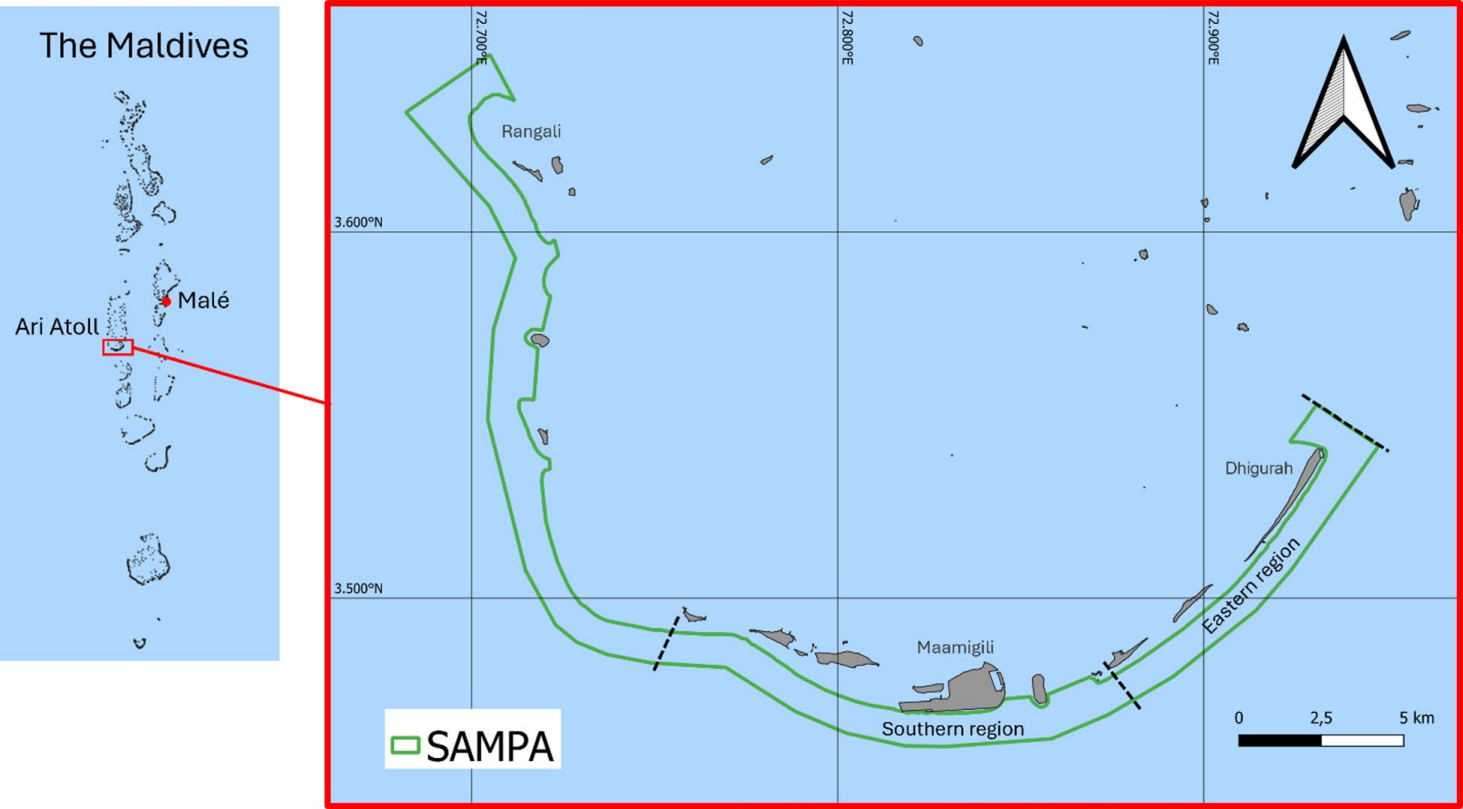
Map of South Ari Maring Protected Area (SAMPA), the Maldives. Dashed black lines denote region boundaries. MPA boundary sourced from UNEP-WCMC and IUCN (2025), island boundaries sourced from OCHA (2024)^51,52^. Maps were generated using QGIS (version 3.4) https://qgis.org/^53^.

This study focused on SAMPA, an MPA which covers the southern tip of Ari atoll, Maldives, encompassing several islands, including inhabited and resort islands. The boundaries of SAMPA extend 1 km from the reef edge to the seaward side from Dhigurah to Rangali (Fig. 1), encompassing an area of 56.1 km^2^^18,36,49,50^. It has previously been noted that whale sharks are most commonly observed in the south of SAMPA during the Northeast Monsoon, while they are observed throughout the area during the Southwest Monsoon^11,41^. To investigate potential drivers of these differences, sightings were subdivided between Southern (≈ 13.6 km²) and Eastern (≈ 11 km²) regions (Fig. 1).

### Environmental variable data compilation

Globally, the distribution of whale sharks has been linked to SST and Chl-a^4^. Chl-a is an indirect index of primary productivity^54^. It is also a proxy for phytoplankton biomass and in turn, zooplankton^55^, a main food source for whale sharks^15–17^.

To investigate the role of environmental variables in driving local seasonal patterns in whale shark sightings, mean monthly Chl-a (mg m⁻³) and mean monthly SST (°C, 11 μm daytime) were obtained as spatial layers from NASA’s Ocean Biology Processing Group for the coordinates encompassing SAMPA (N: 3.66, S: 3.45, E: 73.03, W: 72.63). These data were collected using the Moderate Resolution Imaging Spectrometer Aqua (MODIS-Aqua) at a spatial resolution of 4 km² and temporal resolution of one month^56^. Although daily resolution data were available, they contained a large number of missing values, preventing fine-scale spatial analysis. Therefore, a single Chl-a and SST value for each region per month was determined by calculating the mean value of all raster cells that fell withing the relevant region.

### Sightings collection

Between 2016 and 2019, transect surveys were conducted in the MPA. When weather permitted, surveys were run five days per week (Sunday–Thursday) between 06:30 and 19:00 h. The transect was approximately 24 km in length, starting in Dhigurah and routinely covering the eastern and southern MPA (Fig. 1), while the western area was only sporadically surveyed and excluded from analysis. A 15 m motorised wooden boat followed the transect, with at least two researchers and volunteer assistants recording shark sightings. On sighting, the boat approached sharks and coordinates were recorded^18,44^. The number of sightings per transect survey in each region per month was determined.

### Analysis and software

All analysis was carried out using R (version 4.3.2)^57^, with organisation of data using the *tidyverse* package^58^. Visualisation and mapping were carried out using QGIS (version 3.4)^53^. Generalized additive mixed models (GAMMs) can be used to explore nonlinear relationships between variables, while accounting for repeated measures^59–61^. Using GAMMs, we fit separate models to the data to explore seasonal variation in mean monthly Chl-a, mean monthly SST, and sightings per transect survey in each region per month in relation to environmental variables.

To investigate seasonal variation in environmental variables, two separate GAMMs were fit to mean monthly Chl-a and SST data. The relevant environmental variable was included as the response variable. Month and region (Southern or Eastern) were included as interacting cyclical smooth terms with a main effect of region. Year was included as a random intercept to account for variability among years.

To investigate seasonal variation in shark sightings in relation to environmental variables, monthly shark sightings per survey was included as the response variable. Month and region (Southern or Eastern) were included as interacting cyclical smooth terms. This allowed the seasonal effect to vary between regions. Region was also included as a separate main effect to account for baseline differences in sightings between regions. To investigate potential interactions between environmental variables and whale shark sightings, mean monthly Chl-a and SST were included as smooth terms. To assess the potential for an interaction between Chl-a and SST, a tensor product smooth term for these parameters was included. Year was included as a random intercept to account for variability among years.

All GAMM fitting was carried out using the *gamm()* function from the *mgcv* R package^59^, employing restricted maximum likelihood (REML) for smoothness selection with an unspecified number of basis functions.

## Results

### Seasonal variation in environmental conditions

**Fig. 2:**
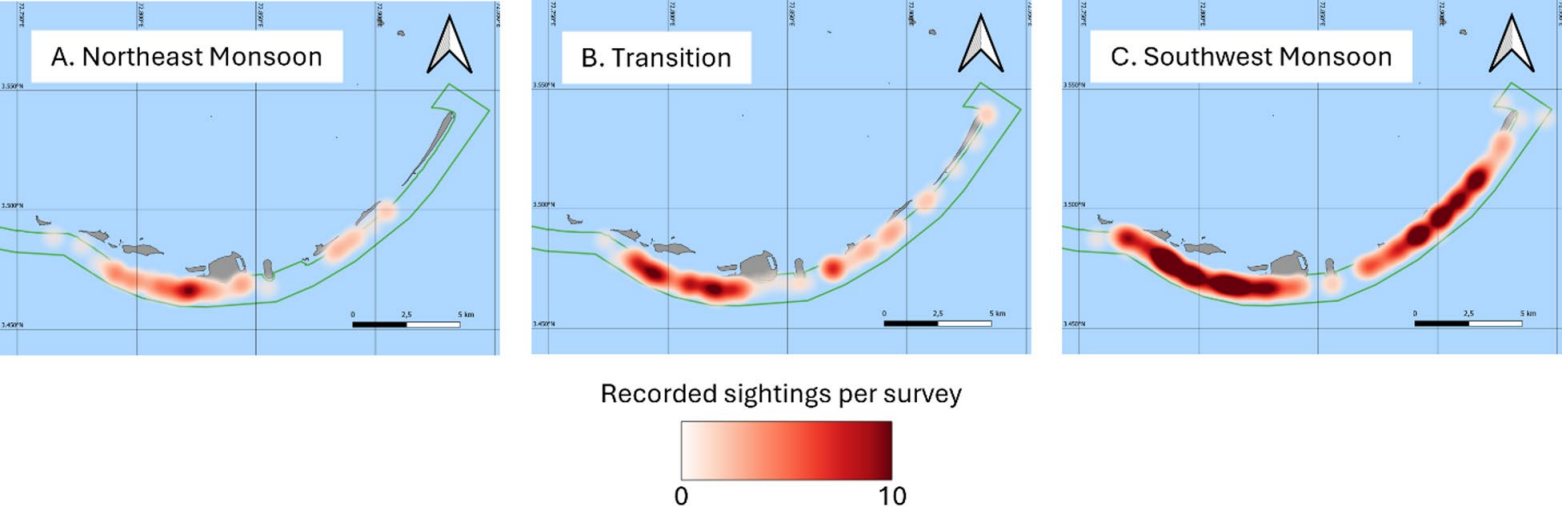
Heat maps of shark sightings in SAMPA. (**A**) Sightings during the Northeast Monsoon. (**B)** Sightings during the transition period. (**C)** Sightings during the Southwest Monsoon. MPA boundary sourced from UNEP-WCMC and IUCN (2025), island boundaries sourced from OCHA (2024)^52,56^. Maps were generated using QGIS (version 3.4) https://qgis.org/^53^.

Between January 2016 and December 2019, 396 transect surveys were carried out, resulting in 451 whale shark sightings (Fig. 2). Sightings per day ranged from 0 to 9 with a mean of 2.1. Mean monthly Chl-a values ranging from 0.1 to 0.63 mg m⁻³ were found over the survey period across both sea regions. A relatively narrow range of mean monthly SST values was found between 28.17 and 31.41 °C.

**Fig. 3:**
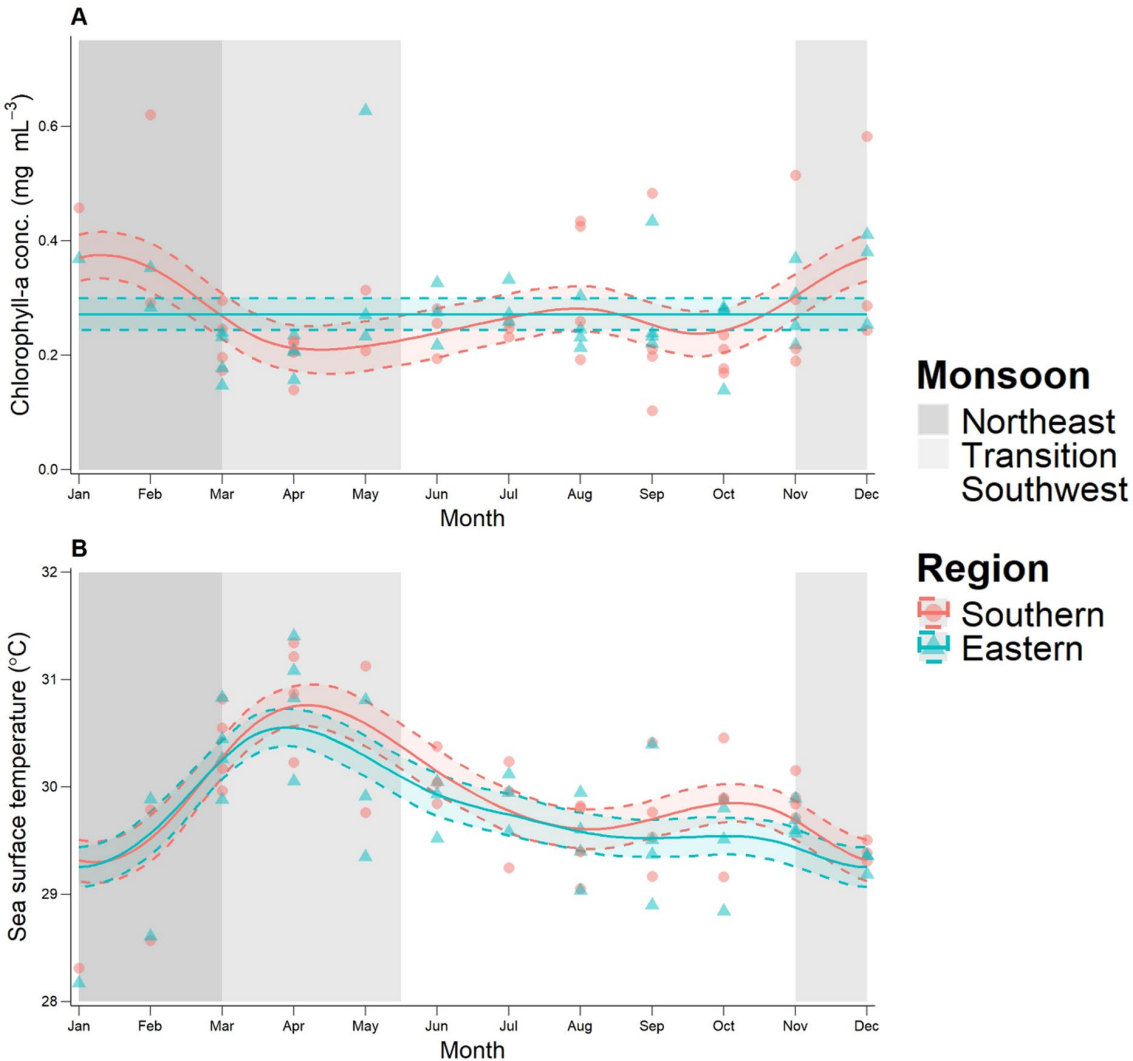
Seasonal variation in environmental variables. (**A**) Mean monthly chlorophyll-a concentration (Chl-a, mg m⁻³) divided between the Southern (red) and Eastern (blue) regions (note difference in seasonal variation between regions). (**B**) Mean monthly sea surface temperature (SST, °C, 11 μm daytime). Solid lines indicate GAMM predictions and dashed lines indicate 95% confidence intervals.

Seasonal dynamics in environmental variables were explored using GAMMs. Significant seasonal variation in Chl-a was found in the Southern region, but not in the Eastern region, suggesting region-specific seasonal dynamics (adjusted R^2^ = 0.116, Table 1; Fig. 3A). Visual examination indicated that Chl-a was higher in the Southern region during the Northeast Monsoon (January to March), with minimal differences between regions during the Southwest Monsoon (mid-May to November, Fig. 3A). GAMM smooth terms indicated seasonal variation in mean monthly SST for both regions, but with little variation between regions (adjusted R^2^ = 0.46, Table 1; Fig. 3B). SST peaked between March and April, during the transition between the Southeast and Northwest Monsoons.

**Table 1:** Outcomes of GAMM fitting for mean monthly Chl-a and SST, including region. An asterisk (*) indicates an interaction. Adj. R^2^ = adjusted R^2^. SE = standard error. EDF = effective degrees of freedom. Ref. DF = reference degrees of freedom.

Main effects					
Model	Region	Intercept	SE	t-value	*p*-value
Chl-a	Southern	0.03 mg m⁻³	0.03 mg m⁻³	9.76	< 0.001
	Eastern	0.03 mg m⁻³	0.02 mg m⁻³	− 0.03	0.977
SST	Southern	29.92 °C	0.09 °C	340.6	< 0.001
	Eastern	29.78 °C	0.11 °C	− 1.32	0.191

Approximate significance of smooth terms					
Model	Term	EDF	Ref. DF	f-value	*p*-value
Chl-a	Region*Southern	4	8	1.83	0.005
	Region*Eastern	< 0.001	8	< 0.001	0.71
SST	Region*Southern	4.61	8	4.3	< 0.001
	Region*Eastern	4.28	8	3.96	< 0.001

### Seasonal variation in whale shark sightings per survey

The GAMM assessing the influence of seasonality and environmental variables on whale shark sightings indicated significant seasonal variation. The Southern region showed more pronounced seasonal variation than the Eastern region, suggesting regional differences in seasonal patterns of occurrence. The Southern region also had a significantly higher estimate for main effect than the Eastern region, indicating a higher overall number of sightings per survey. Number of sightings were significantly associated with mean monthly Chl-a. Sea surface temperature (SST) was not found to be significantly associated with the number of sightings. The interaction between Chl-a and SST was not found to have a significant effect on number of sightings (Table 2).

**Table 2:** Outcomes of GAMM fitting for shark sightings per survey area, including environmental variables and region. An asterisk (*) indicates an interaction. Adj. R^2^ = adjusted R^2^. SE = standard error. EDF = effective degrees of freedom. Ref. DF = reference degrees of freedom. te = tensor flow product.

Main effects				
Region	Intercept	SE	t-value	*p*-value
Southern	1.52	0.19	8.12	< 0.001
Eastern	0.79	0.19	− 3.78	0.977

Approximate significance of smooth terms				
Term	EDF	Ref. DF	f-value	*p*-value
Region*Southern	2.91	8	1.55	0.004
Region*Eastern	1.96	8	0.68	0.045
CHL	1	1	6.25	0.014
SST	1	1	1.46	0.232
te(CHL, SST)	1	1	0.61	0.438

**Fig. 4:**
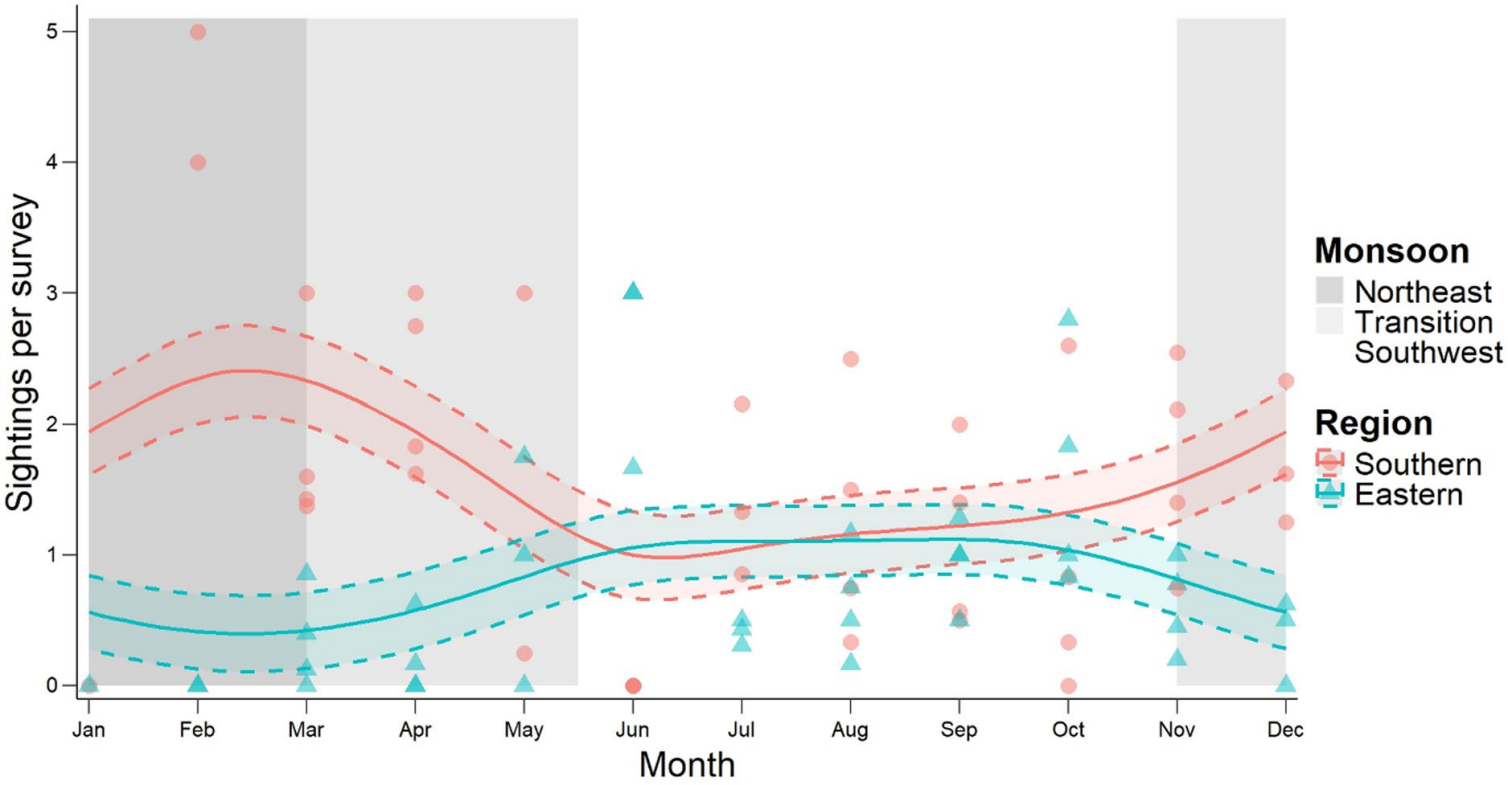
Whale shark sightings per survey. Sightings are divided between the Southern (red) and Eastern (blue) regions. Note that the increase in sightings in the Southern region during the Northeast Monsoon (January to March) coincided with the annual peak in mean monthly Chl-a. Solid lines indicate GAMM predictions and dashed lines indicate 95% confidence intervals.

Visual examination of model predictions suggested mean monthly sightings in the Southern region peaked towards the middle of the Northeast Monsoon (January to March) and gradually declined over the transition between the Northeast and Southwest Monsoons (April and early May, Fig. 4). The increase in shark sightings in the Southern region during the Northeast Monsoon coincided with the peak in Chl-a (Fig. 3A). Sightings in the Eastern region were notably low during the Northeast Monsoon. Sightings were approximately equally divided between the two regions during the Southwest Monsoon (mid-May to November).

## Discussion

SAMPA is one of very few year-round aggregations of resident whale sharks in the world^18,23,45^. Many other year-round aggregations are influenced by human activities, such as provisioning of food or the use of bright lights to attract prey (light pooling), which are not permitted in SAMPA^23,36,50,62^. As such, SAMPA is well-suited for the study of natural local seasonal dynamics in whale shark distribution. While both Chl-a and SST showed significant seasonal variation in the study area, only Chl-a differed significantly between SAMPA regions. A pronounced peak in Chl-a was observed in the south of the MPA during the Northeast Monsoon. This is likely the result of prevailing winds^48^. Shark sightings were found to be significantly associated with Chl-a and the annual peak in Chl-a in the south of the MPA coincided with the peak in shark sightings per survey. Seasonal rhythms persisted despite a reported decline in overall whale shark abundance over the survey period^18^.

The association between shark sightings and Chl-a suggests that primary production, and associated zooplankton biomass^55^, is likely a key driver of whale shark abundance at the local level within the year-round aggregation. Previous work has linked whale shark abundance to Chl-a, zooplankton biomass, and SST in seasonal aggregations^27–29,35,37,38^, including at the ocean scale^4,34^. Similar associations between Chl-a and other filter-feeding megafauna, such as the reef manta ray (*Mobula alfredi*), have been reported^63^. Such broad-scale analysis requires comparing sea areas with highly variable environmental conditions as well as varying degrees of human influence and protection^4,34^. Given the homogeneity of management across the study site, and the lack of regional variation in SST observed in SAMPA, the present study was able to isolate Chl-a as a potential key driver of abundance. Water temperature conditions throughout SAMPA are likely uniformly suitable for whale sharks, allowing them to selectively feed in areas with abundant food. This supports the hypothesis put forward by Anderson and Ahmed (1993), and more recently by Valsecchi et al. (2021), that whale shark abundance within SAMPA is driven by prey abundance^11,41^. It also supports the views of Afonso et al. (2014) that, while whale shark abundance globally is largely driven by abiotic factors such as SST, local patterns are more often driven by prey abundance^38^. Future research should aim to validate this through direct sampling of zooplankton abundance and community composition across seasons throughout the MPA. Furthermore, these studies could incorporate additional physical parameters such as salinity and dissolved oxygen to further examine how zooplankton distribution patterns influence sightings of whale sharks within the MPA.

Our findings help define preferred whale shark habitats based on SST and Chl-a values. This information may help to predict aggregations elsewhere^34,35^, with validation possible through targeted surveys, satellite imagery^64,65^, or citizen science platforms like the Big Fish Network (BFN)^66^. Establishing these environmental relationships also provides a baseline which could be used to predict how future climate-driven changes in ocean conditions may influence the stability of whale shark aggregations^4^.

Whale sharks have been protected in the Maldives since 1995, making them the first shark species in the country to receive this status^67^. Initially protected under the Fisheries Act (Act No. 14/2019), they were officially uplisted in 2023 under the Environmental Protection and Preservation Act (Act No. 4/93) and are now included in the Ministry of Environment’s Protected Species List^68^. Within SAMPA, whale sharks are protected both through protected area regulations and species-specific regulations (e.g., restrictions on vessel numbers and speed limits around whale sharks). Despite these protections, vessel strikes and the cumulative effects of repeated tourism interactions are recognised as ongoing pressures on whale sharks in the Maldives and elsewhere^18,22,36^. The present study demonstrates that whale shark distribution within SAMPA is spatially heterogeneous and characterised by predictable seasonal shifts in areas of high use. These patterns represent important ecological features of the MPA that are not explicitly accounted for in current management or enforcement practices. Consideration of this spatio-temporal structure could inform how, when, and where policing of existing regulations are prioritised within the MPA in line with emerging practice of dynamic area-based management for mobile marine megafauna^13,69^.

Following the release of the SAMPA management plan in February 2025, patrols led by MPA rangers commenced. While these currently rely on monitoring vessel traffic across the MPA, the findings of this study provide an empirical basis for a more targeted, data-driven approach that aligns patrol effort with predictable patterns in whale shark occurrence. Such a dynamic strategy could improve the efficiency of monitoring and enforcement efforts by focusing limited resources on locations with a high abundance of sharks. In addition, targeted awareness sessions for tourism operators could be planned prior to seasonal peaks in whale shark activity near their bases (e.g., for resorts or local island-based dive and excursion centres).

Surveys could not be carried out during unfavourable weather conditions, meaning survey effort was not equal throughout the year^18,44^. To account for this, data were analysed as sightings per survey, rather than as raw counts. This means that we are lacking information on shark behaviour during particularly windy days. In future, many of the challenges associated with boat-based surveys could be overcome using Uncrewed Aerial Vehicles (UAVs)^70–72^ or detection of whale sharks from satellite images^64,65^. While UAVs are also limited by being unable to fly in high winds and satellites are limited by cloud coverage, integration of diverse data collection methods could potentially allow for finer-scale temporal analysis.

MODIS-Aqua data provides a long-standing and widely used measure of Chl-a concentration which has been integrated into many ecological studies^40,73^. The presence of missing values across both time and region presented a challenge during analysis^56^. To overcome this challenge, we maximised data coverage by pooling environmental variables across months and regions. Averaging Chl-a values may have resulted in the loss of information on extreme upper or lower values which could provide additional insight into local movements. The range of Chl-a we report (0.1 to 0.63 mg m⁻³) falls in the lower end of the range reported for the whale shark aggregation in Saleh Bay, Indonesia, during a single season (0.2 to 3.45 mg m⁻³)^40^. This could potentially be the result of averaging. Importantly, however, previous studies have not investigated the range of conditions experienced by whale sharks year-round. Our methods were appropriate for investigating seasonal rhythms, which occur over the course of months, and were sufficient to detect a positive association between shark sightings per survey and Chl-a concentration. In future work, finer-scale temporal patterns in shark abundance could be investigated by integrating multiple other satellite data sources (e.g., the Visible Infrared Imaging Radiometer Suite (VIIRS) sensor aboard the NOAA-20 satellite) or predictive modelling to fill in missing values in environmental variables^73^.

Whale sharks are thought to spend a substantial (> 30%) amount of time at the ocean surface in order to feed and thermoregulate^74–76^. Sightings in the present study were tightly linked to this surface behaviour and possibly biased towards individuals carrying out those behaviours. Other behaviours, such as resting and transit, may occur in deeper waters beyond the MPA and are not represented in the present study. The SAMPA whale shark aggregation is composed primarily of juvenile males, reflecting age-class and sex specific aggregations commonly observed in other sites^18,24,44^. Given the scarcity of females and older animals in such aggregations, it seems likely that they have substantially different physiological needs. As a result, the findings of this and other studies of the drivers of abundance^38,40^ should not be generalised to the species as a whole. Future work could assess whether the patterns described in the present study hold true for other age- and sex-classes^5,23^. As whale sharks have unique markings, drivers of individual-level behaviour could also be explored in future work^18,44,77^; however, this would limit data to identified individuals.

## Conclusion

We have conducted the first quantitative analysis of local seasonal variation in whale shark abundance in a year-round aggregation. Within an MPA, mean monthly Chl-a was significantly associated with the number of shark sightings. This suggests that whale shark abundance is driven by prey availability on a local level. These findings support reported links between Chl-a and whale shark abundance in seasonal aggregations elsewhere. Defining habitat and the drivers of whale shark abundance could provide insight into the potential impact of ecosystem changes on this and other marine megafauna species.

## Supplementary Information

Below is the link to the electronic supplementary material.

Supplementary Material 1

## Data Availability

All data generated or analysed during this study are included in this published article and Supplementary_Information_1.zip. Data and code are available at Zenodo.org: https://doi.org/10.5281/zenodo.15755924. MODIS-Aqua Level-2 ocean colour data used in this study are publicly accessible through NASA’s Ocean Biology Processing Group (OBPG) data portal and can be downloaded via NASA’s open data policy (https://science.nasa.gov/researchers/science-information-policy/).
